# Biomarkers, Proteoforms, and Mass Spectrometry–Based Assays for Diabetes Clinical Research

**DOI:** 10.1210/clinem/dgaf159

**Published:** 2025-03-08

**Authors:** Lorenz A Nierves, Tai-Tu Lin, Annie Moradian, Qingqing Shen, Salvatore Sechi, Michael J MacCoss, Jun Qu, Jennifer E van Eyk, Andrew N Hoofnagle, Wei-Jun Qian

**Affiliations:** Translational Omics, Biological Sciences Division, Pacific Northwest National Laboratory, Richland, WA 99354, USA; Translational Omics, Biological Sciences Division, Pacific Northwest National Laboratory, Richland, WA 99354, USA; Precision Biomarker Laboratories, Cedars-Sinai Medical Center, Los Angeles, CA 90048, USA; Department of Pharmaceutical Sciences, University at Buffalo, Buffalo, NY 14214, USA; Division of Diabetes, Endocrinology, and Metabolic Diseases, National Institute of Diabetes and Digestive and Kidney Diseases, National Institutes of Health, Bethesda, MD 20892, USA; Department of Genome Sciences, University of Washington, Seattle, WA 98195, USA; Department of Pharmaceutical Sciences, University at Buffalo, Buffalo, NY 14214, USA; Precision Biomarker Laboratories, Cedars-Sinai Medical Center, Los Angeles, CA 90048, USA; Smidt Heart Institute, Cedars-Sinai Medical Center, Los Angeles, CA 90048, USA; Department of Laboratory Medicine and Pathology, University of Washington, Seattle, WA 98195, USA; Translational Omics, Biological Sciences Division, Pacific Northwest National Laboratory, Richland, WA 99354, USA

**Keywords:** proinsulin, glucagon, pancreatic dysfunction, TaMADOR, type 1 diabetes, post-translational modification

## Abstract

The prevalence of diabetes, particularly type 2 diabetes, has reached epidemic proportions globally. The number of patients with type 1 diabetes (T1D) is also increasing rapidly. Despite advancements in understanding the pathogenesis of diabetes, the lack of circulating pancreatic biomarkers and reliable clinical-grade assays remains a major gap in diabetes research, often hindering the ability to adequately assess disease progression and therapeutic responses. This mini-review discusses emerging pancreatic biomarkers, with an emphasis on T1D, the limitations of current immunoassays, and the expanding role of mass spectrometry–based assays. Highlights include the recent work within the NIDDK-funded “Targeted Mass Spectrometry Assays for Diabetes and Obesity Research (TaMADOR)” consortium, which aims to develop robust, quantitative, and transferable assays for translational research. The review also emphasizes the importance of proteoform-specific assays for monitoring pancreatic function, including prohormone processing during disease progression or in responses to therapy.

Diabetes mellitus encompasses a group of metabolic disorders that arise when the body cannot properly regulate blood glucose levels, due to either inadequate insulin secretion, or insulin resistance, or both. Both diabetes and obesity—a major risk factor for diabetes—have reached epidemic levels (1). The rise in diabetes cases is primarily due to the increase in type 2 diabetes (T2D); however, there has also been a significant global increase over the past several decades in the incidence of type 1 diabetes (T1D)—a chronic disorder caused by the autoimmune destruction of pancreatic β-cells (2). Despite the etiological differences of the various forms of diabetes, a key common feature is the presence of pancreatic dysfunction, which may involve issues with endocrine hormone secretion, exocrine digestive enzyme secretion, or both. For instance, the loss of functional β-cells is well recognized as a key pathophysiological mechanism of both T1D and T2D (3).

Clinical research in diabetes focuses on disease prevention, effective long-term disease management, and the development of new treatments, with the ultimate goal of finding a therapeutic cure. Circulating biomarkers that reflect pancreatic endocrine and exocrine functions could play a crucial role in translational research, as they offer insights into physiological processes, the functional state of the cells, or pathological conditions (4). Such biomarkers have potential applications in diagnosis, prognosis, monitoring treatment responses, and tracking disease progression. Currently, however, diabetes research depends on several circulatory biomarkers such as glucose, HbA1c (glycated hemoglobin), C-peptide, and autoantibodies (AABs), which are inadequate for capturing the complex pathophysiology involved in diabetes (5). The lack of additional mechanistic biomarkers severely restricts the ability to accurately assess disease progression and therapeutic responses. Moreover, reliable and accurate clinical-grade assays for measuring these biomarkers are also essential in translational research. Therefore, there remains a pressing need for validated blood biomarkers, especially those reflecting pancreatic exocrine and endocrine function (eg, β-cell function), as well as standardized assays for these biomarkers in translational diabetes research.

In this mini-review, we will discuss emerging circulating biomarkers with a focus on pancreatic endocrine and exocrine protein/peptide markers in T1D research, the fundamental limitations of current immunoassays, and the expanding roles of mass spectrometry (MS)-based assays in clinical research. In light of the 75th anniversary of the National Institute of Diabetes and Digestive and Kidney Diseases (NIDDK) in 2025, we highlight recent progress within the “Targeted Mass Spectrometry Assays for Diabetes and Obesity Research (TaMADOR)” consortium. This research program, supported by NIDDK, aims to enhance the rigor and reproducibility in diabetes clinical research. TaMADOR, as a collaborative effort, was conceived to develop robust, quantitative, transferable, and harmonizable assays for the quantification of proteins and peptides of interest to the diabetes research community.

## Emerging Pancreatic Biomarkers

Current biomarkers used in clinical practice for diabetes, including glucose, hemoglobin A1c (HbA1c), insulin, C-peptide, and autoantibodies (AABs), are mainly diagnostic markers with limited prognostic value (5). For example, AABs against specific β-cell antigens (eg, insulin) serve as signals of autoimmunity and in most cases provide a relatively good risk assessment for the eventual development of T1D; however, they have only limited values in predicting the rate of disease progression (6). Pancreatic β-cell function in patients is currently assessed by measuring plasma glucose, insulin, and C-peptide concentrations in the fasting state or as part of a glucose tolerance test (4). Despite substantial advances in our understanding of the pathogenesis of both T1D (7) and T2D (8) during the past few decades, there remains a significant lack of blood-based biomarkers that can reflect pancreatic function—especially β-cell function, stress, and β-cell mass—and predict disease progression or monitor therapeutic responses. The challenges in the discovery of novel blood biomarkers are due, in part, to the nature of targeting cell populations, such as pancreatic β-cells, which comprise only ∼.002% of body mass (9). Nevertheless, several novel pancreatic biomarkers, including exocrine and endocrine markers (Table 1), have been reported to have promising clinical significance.

**Table 1.** Emerging pancreatic biomarkers with clinical utilities

Biomarkers	Clinical significance
*Exocrine markers*	
Pancreatic α-amylase	Biomarker for monitoring exocrine function (10, 11)
Lipase	Biomarker for monitoring exocrine function (10) Serological marker for improving disease staging in T1D (12)
Trypsin(ogen)	Lower level in patients with recent T1D diagnosis (13) Serological marker for improving disease staging in T1D (12)
*Endocrine markers*	
Insulin/C-peptide	Measurements for assessing β-cell responses, insulin sensitivity, and β-cell function (4)
Proinsulin	Biomarkers of β-cell dysfunction or stress in T2D (14) and T1D (15) Elevated ratios of proinsulin to C-peptide in new onset T1D (16) Potential utility in predicting disease progression in at risk individuals for T1D (17).
ProIAPP	Elevated ratios of proIAPP_1-48_ to IAPP in T1D subjects (18).
Glucagon and proglucagon-derived peptides	Biomarker for α-cell dysregulation in both T1D (19) and T2D (20).
Chromogranin A	Levels rise with progression of T1D (21) Potential therapeutic marker in T1D (22)

Functional defects in the exocrine pancreas have been increasingly recognized in both T1D and T2D (7, 23). In many cases, dysfunction of the exocrine pancreas is considered a specific form of diabetes (24). As a result, the exocrine enzymes lipase and amylase have been suggested as key functional biomarkers in patients with T1D, T2D, or metabolic syndrome (10, 11). Similarly, serum lipase and trypsinogen have been reported as serological markers for disease staging in T1D (12). A recent proteomic discovery study using monozygotic twins discordant for T1D identified 5 other exocrine proteins as promising biomarkers for T1D: chymotrypsinogen B, chymotrypsinogen B2, trypsinogen 1, trypsinogen 2, and carboxypeptidase B1 (25). Furthermore, the levels of trypsin(ogen) were screened in >1000 samples from well-characterized individuals with autoimmune diabetes using the Delfia Neonatal IRT assay kit (PerkinElmer), which measures a mixture of different forms of trypsin/trypsinogen. The results demonstrated that trypsin(ogen) levels are lower before, at, and after diabetes diagnosis (25).

Since the endocrine function of pancreatic islets plays a central role in all forms of diabetes, much effort has been made in identifying endocrine biomarkers that can reflect or predict islet cell function. Hormones such as insulin, glucagon, and islet amyloid polypeptide (IAPP) are first synthesized as larger precursor proteins. They are then subjected to multi-step post-translational modifications, including proteolytic processing, resulting in the final mature, biologically active peptide hormones (26, 27). Islet prohormone processing has become an emerging area of research in diabetes and prohormone processing defects have been reported in both T2D (28) and T1D (15). For example, elevated proinsulin-to-insulin or proinsulin-to-C-peptide ratios have been reported as biomarkers of β-cell dysfunction in T2D (14) and T1D (17) with potential value in disease prediction. The complexity of islet prohormone processing illustrates the potential importance of measuring specific proteoforms as biomarkers, and the need for proteoform-specific assays.

### Proteoforms as Biomarkers

**Figure 1.**
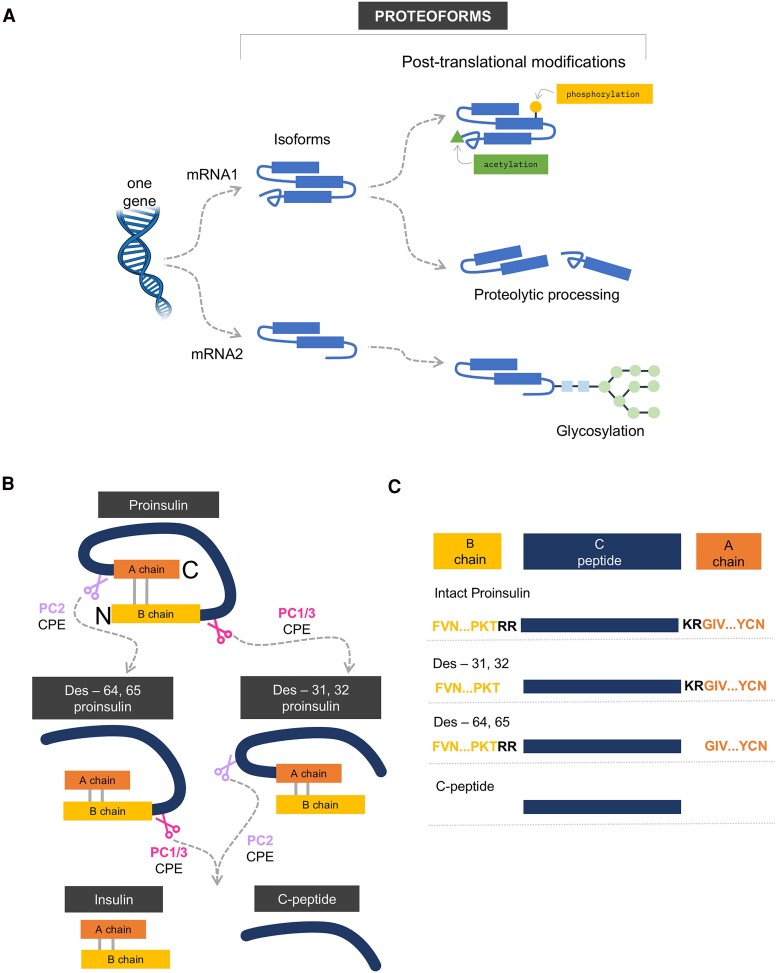
A, The general concept of proteoforms. B, Processing of proinsulin. The intact proinsulin following signal peptide removal can be partially cleaved, leading to 2 intermediate proteoforms— des-31,32 and des-64,65 proinsulin. The intermediate proteoforms can be further processed into mature insulin and C-peptide. C, Sequence alignments of insulin proteoforms, illustrating the subtle differences between proteoforms. The junction residues between C-peptide and A-chain/B-chain are highlighted in black bold letters.

Figure 1A illustrates the general concept of “proteoform,” which is used to describe a specific molecular form of a protein product arising from a specific gene (29). A single gene can be spliced into multiple mRNAs resulting in several isoforms. Each isoform can be further processed into many different proteoforms through post-translational modifications, including proteolytic processing (29). Figure 1B illustrates the processing steps of proinsulin that occur within the secretory granules of pancreatic β-cells. Proinsulin is subjected to initial proteolytic cleavage by preprotein convertases (PC) (27). In one pathway, proinsulin is cleaved by PC1/3 after residue 32 and the dibasic residues 31 and 32 (2 arginines, RR) are then removed by carboxypeptidase E, resulting in the intermediate des-31,32 proinsulin form. In another pathway, proinsulin is cleaved by PC2 after residue 65 and the dibasic residues 64 and 65 (lysine and arginine, KR) are then removed by carboxypeptidase E, leading to the intermediate form des-64,65 proinsulin. The 2 intermediate forms are then further processed into mature insulin and C-peptide. In summary, after the translation of the insulin gene, proinsulin can be post-translationally processed into 4 additional proteoforms. Due to the amino acid sequence similarity between different proteoforms, it is often challenging to develop assays specific to each proteoform. To fully evaluate the underlying mechanisms of proinsulin processing and the potential utility of proinsulin proteoforms as mechanistic biomarkers, it will be critical to develop highly specific and reliable assays for multiple proteoforms including intact proinsulin, des-31,32 proinsulin and des-64,65 proinsulin. The performance characteristics of current commercial immunoassays for detecting intact proinsulin (30), and total proinsulin (which includes intact and partially processed proinsulin forms) (31, 32) have not been rigorously established (33).

Despite the limitations of the current assays, the clinical significance of quantifying various proinsulin proteoforms is compelling. For example, circulating proinsulin to C-peptide ratios were observed to be elevated in individuals with new or recent onset T1D (16, 34), and were also demonstrated to predict disease progression in individual at risk for T1D (17, 35-37). These reports illustrate the potential of the proinsulin to C-peptide ratio as an indicator of β-cell dysfunction and stress in T1D. Another promising endocrine biomarker is IAPP (also known as amylin) and its proteolytic proteoforms. Like proinsulin processing, proIAPP post-translational processing results in proIAPP_1-67_, proIAPP_1-48_, non-amidated IAPP, and mature IAPP proteoforms (38). Plasma IAPP levels were observed to be higher in young individuals with incident T1D (39). Moreover, the ratio of proIAPP_1-48_ to IAPP was shown to be significantly elevated in participants with T1D, as well as islet transplant recipients, by using a proIAPP_1-48_ immunoassay (18). However, it should be noted that the robustness of the immunoassay for proIAPP has not yet been fully validated.

Besides markers of β-cell dysfunction, α-cell dysregulation and its roles in T2D (20) and T1D (19) have also been recognized. Glucagon has traditionally been recognized as a counter-regulatory hormone to insulin but is increasingly recognized as an important marker for α-cell dysregulation (19, 20). Moreover, proglucagon has its own complex prohormone processing pathway, which results in distinct proteoforms (eg, glucagon, oxyntomodulin, glucagon-like peptide [GLP]-1, and GLP-2) (40). The significance of proglucagon-derived peptides, such as GLP-1 and GLP-2, in the regulation of metabolism and feeding is well recognized (41, 42); however, the roles of specific proglucagon proteoforms in the pathophysiology of diabetes are still largely underexplored. Finally, chromogranin-derived peptides may also have a role in diabetes (43). For example, serum chromogranin A (CHGA) has been observed to continually rise during the progression of T1D (21) and has been proposed as a therapeutic marker to monitor the effects of verapamil treatment in T1D (22).

## Immunoassays and Their Limitations

Many of the current assays for protein and peptide biomarkers that are used in the diagnosis of diabetes and in clinical diabetes research, such as C-peptide (44), insulin (45), HbA1c (46), and AABs (47), are immunoassays, which solely rely on antibody reagents. It is well recognized that immunoassays have several fundamental drawbacks (48), including a lack of concordance across platforms, potential interference or cross-reactivity with endogenous and exogenous substances (49), a lack of specificity and reproducibility for targeted proteoforms (particularly when polyclonal antibodies are used), and issues caused by AABs and anti-reagent antibodies. This impacts both patient care and clinical research. For example, current C-peptide immunoassays are sensitive, but concerns have been raised regarding cross-reactivity with proinsulin, and poor inter-platform concordance (50, 51). In addition, despite efforts to address the issue, the lack of standardization of current insulin immunoassays is acknowledged as a significant barrier to achieving meaningful treatment guidelines (52). Beyond biomarkers in diabetes, some commonly applied immunoassays have failed to detect their intended targets, leading to false conclusions (53-56). For example, the commercial enzyme-linked immunosorbent assay (ELISA) for the pancreatic biomarkers, zona pellucida-like domains protein 1 (CUZD1), has been found to mistakenly recognize CA125, a known cancer antigen, which is nonhomologous to its intended target (53). Hence, there is a significant need for validation and standardization of immunoassays for current diabetes biomarkers and emerging biomarkers. Advances in MS-based assays hold promise for addressing these challenges, as the field moves toward reliable and accurate biomarker measurements.

## Mass Spectrometry–Based Assays and TaMADOR

Protein/peptide quantification by liquid chromatography–tandem mass spectrometry (LC-MS/MS) is now recognized as a gold-standard technology capable of providing the foundation for robust, quantitative, and standardizable assays. While antibodies may be used during sample preparation, the specificity of LC-MS/MS as a detection method is antibody-independent and allows for the verification of the exact amino acid sequence of each analyte. Targeted MS techniques, including selected reaction monitoring (SRM) (also known as multiple reaction monitoring, MRM) (57, 58) and parallel reaction monitoring (PRM) (59, 60), directly detect analytes by using finely tuned electric fields. When these protein analytes remain intact during sample preparation, targeted MS identifies individual proteoforms with incredible specificity. When protein analytes are proteolyzed during sample preparation, surrogate peptides are used to quantify families of proteoforms that share those specific peptides. As a bonus of this “bottom-up” approach, proteolysis eliminates common assay interferences, including AABs, anti-reagent antibodies, and other polypeptides, which can interfere with immunoassays or antibody-based enrichment/sample preparation. Thus, targeted MS assays can overcome many limitations of immunoassays or can alternatively serve as arbiters in standardization efforts (61). One clinical example of MS overcoming the limitations of immunoassays is in the measurement of serum concentrations of thyroglobulin, used to evaluate the effectiveness of treatment and the recurrence of thyroid cancer (62). The thyroglobulin immunoassay can be affected by anti-thyroglobulin AABs, resulting in falsely negative thyroglobulin measurements (63). By using targeted LC-MS/MS coupled with surrogate peptide immunocapture, the quantification of thyroglobulin-specific peptides avoids the interference of AABs and anti-reagent antibodies (63, 64).

MS-based assays also play a major role in assay standardization efforts. A notable example is the NIDDK-supported C-peptide standardization program (51), which has demonstrated the critical value of the LC-MS reference method in enhancing concordance among various methods and laboratories. The initiative also presents an opportunity for commercial immunoassay manufacturers to improve their products. The measurement of plasma insulin-like growth factor 1 (IGF-1) is another example where poor agreement between platforms and a lack of consistency between laboratories were observed (65). An interlaboratory comparison of an LC-MS/MS-based IGF-1 assays across 5 laboratories using 5 different instruments (66) achieved a mean imprecision of 11.1% when a single-point calibration approach was used. These efforts demonstrated the potential of MS-based assays for clinical applications and for serving as a reference method in assay standardization.

As part of the National Institutes of Health (NIH) initiative to enhance rigor and reproducibility in scientific research (67), the NIDDK has launched a mini-consortium called TaMADOR (https://panoramaweb.org/TAMADOR/project-begin.view). This consortium aims to address the need for reliable clinical-quality assays to enhance the reproducibility of diabetes clinical research through the development of robust, transferable, harmonizable targeted MS assays for the quantification of proteins and peptides of interest (eg, C-peptide, insulin, glucagon) to the diabetes translational research community. Additionally, the consortium aims to develop assays that can account for the various proteoforms (eg, proinsulin, proglucagon, and proIAPP) generated during prohormone processing, which might serve as biomarkers of disease prognosis and help monitor therapeutic responses. To ensure reproducibility, all monoclonal antibodies developed within TaMADOR will be deposited into the Developmental Studies Hybridoma Bank, a national resource established by the NIH. Highlighted below are some of the major ongoing efforts of this program.

### An Antibody-Free Multiplexed Insulin and C-Peptide LC-MS/MS Assay

**Figure 2.**
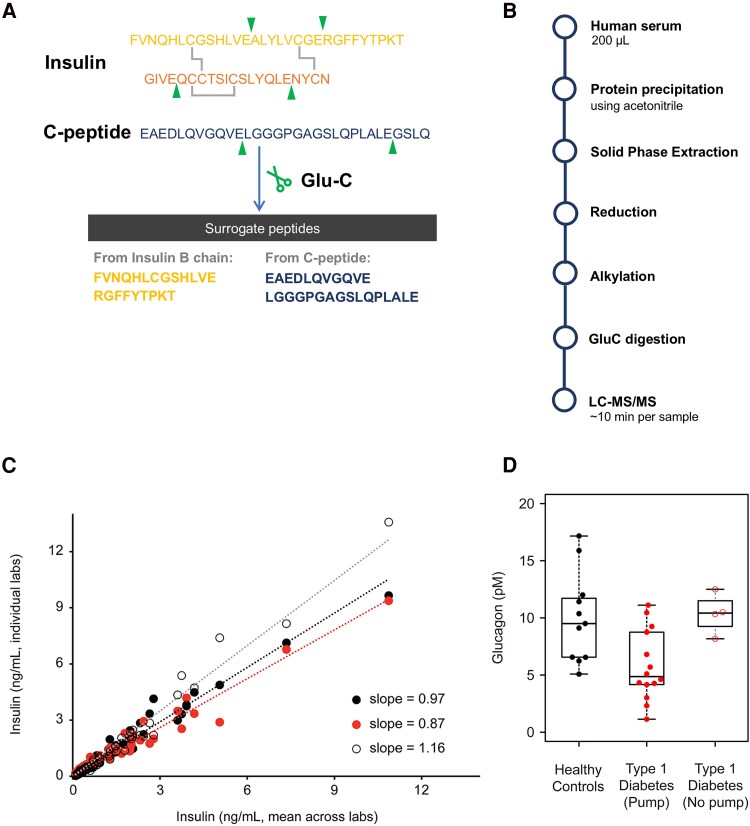
A, Surrogate peptides from insulin and C-peptide generated by Glu-C digestion. B, Sample processing workflow of the LC-MS/MS assay for insulin and C-peptide using a 96-well format. C, Interlaboratory comparison of the LC-MS/MS insulin assay. The concentration observed for each sample is plotted for each laboratory vs the mean concentration observed across all 3 laboratories. The data from each laboratory is represented by individual types of circles. The slopes of each linear regression are listed (dotted lines). D, Fasting plasma glucagon in patients with T1D and controls as measured by the LC-MS/MS glucagon assay. Fasting plasma concentrations of glucagon observed for patients with T1D treated with an insulin pump or injectable insulin (No pump) are compared with those observed in fasting healthy controls. The figure panel C is reproduced from Moradian et al (71).

Due to the poor concordance of commercial immunoassays for insulin (52) and C-peptide (51), and the legitimate concern for cross-reactivity from related circulating peptides such as proinsulin proteoforms, it is desirable to develop a multiplexed MS assay that can obviate the need for antibodies and can be transferred to clinical laboratories with available standard equipment. Previously developed assays, including the reference measurement procedures, relied on the detection and quantification of intact insulin and C-peptide (68, 69). However, these assays exhibit poor sensitivity due to the low ionization efficiency of the lengthy peptides. To achieve the necessary sensitivity, these assays relied on antibodies or two-dimensional chromatography, each of which introduced potential complications from autoantibodies or other interferences, or limited throughput. To address the poor ionization of intact insulin and C-peptide, and to enhance sensitivity, we explored the use of endoproteinase GluC. Since C-peptide does not contain K or R residues, which are cleavage sites for the typical MS protease trypsin, GluC was more appropriate as it cleaves on the carboxyl-side of the acidic residues, glutamic acid and aspartic acid. By testing different digestion conditions, we were able to reproducibly liberate 2 shorter surrogate peptides from C-peptide (EAEDLQVGQVE and LGGGPGAGSLQPLALE) in ammonium bicarbonate buffer after the precipitation of bulk proteins with acetonitrile (70). Similarly, 2 peptides were liberated with Glu-C proteolysis that proved to be sensitive and specific (RGFFYTPKT and FVNQHLCGSHLVE) for insulin (Fig. 2A). We then developed a multiplexed LC-MS/MS assay for C-peptide and insulin with a relatively simple sample preparation workflow (Fig. 2B) consisting of protein precipitation, Glu-C digestion, and LC-MS/MS (72). The assay was determined to be linear and precise, although interference due to hemolysis was noted. The combination of insulin and C-peptide in a single assay permits a more complete assessment of β-cell function in patients.

One of the benefits of MS-based assays is the ability to directly quantify analytes, but it is also possible to make assays traceable to reference materials, reference measurement procedures, and ultimately SI (molar) units with the help of adequate external calibrators. To this end, the TaMADOR consortium worked with the C-peptide standardization program, administered by the University of Missouri, to establish a secondary calibration material of assigned value that could be used to ensure accuracy (73). Similarly, the same secondary calibrator was characterized with the National Metrology Institute of Japan certified reference material (NMIJ CRM 6209)—a primary reference material—that could enable accurate insulin quantification. As a result, specific and accurate quantification of both analytes has been made possible with a single assay.

One of the goals of the TaMADOR consortium is the transfer of quantitative targeted assays between laboratories. This has traditionally been a challenge for biomedical research in general, with immunoassays identified as a major source of the problem. One of the challenges of interlaboratory measurements of LC-MS/MS assays is the usage of different LC-MS/MS systems by the participating laboratories. Measures were taken to align all aspects of the method comparison, including calibration, a highly detailed standard operating procedure, and the experimental design/plate layout. Each laboratory independently verified good precision, linearity, and analytical sensitivity, indicating that the method performs well across different systems. As a result, the biases observed between the laboratories likely stem from the differences between LC-MS/MS configuration and sample preparation. These findings should guide assay developers in considering calibration approaches to enhance comparability and accuracy in their own laboratories. Interlaboratory comparison of the LC-MS/MS assay also required calibration standards. As observed for other assays, the use of a matrix-matched single-point calibrator effectively reduced imprecision of the assay over time. The benefits of using a human-derived sample for the calibration of the assay include the removal of the variability inherent in the generation of the calibration curve and instability or adsorption of pure peptide/protein over time. By including replicates of this pooled human plasma in each batch at each site, we were able to minimize variability of analysis across batches and sites. In the end, median imprecision across the 3 laboratories was observed to be 10.8% and 15.3% for C-peptide and insulin, respectively, when using a single-point calibrator (Fig. 2C) (71). The strong agreement between the 3 TaMADOR laboratories and the robust correlation with this reference method support the idea that such standardization programs can make LC-MS/MS-based assays highly accurate, precise, and transferable for clinical research and patient care.

The combined multiplex targeted assay of insulin and C-peptide was recently used in a clinical study of the influence of glucagon on the secretion and hepatic clearance of insulin (74). In that study, the traceability of the measurement of C-peptide and insulin to SI units (ie, molar concentrations) allowed for the direct, simultaneous evaluation of β-cell release of each analyte, which is equimolar, and the subsequent degradation, presumably by hepatic clearance, of insulin. The results demonstrated that glucagon slows the degradation of insulin in vivo, a previously unrecognized function of α-cells.

### Developing MS Assays for Proinsulin and Proglucagon Proteoforms

As mentioned above, another major focus of TaMADOR is to develop novel proteoform-specific assays that can specifically target islet prohormone proteoforms (eg, from proinsulin and proglucagon) as potential novel biomarkers of interest in T1D (15). These novel assays will enable a detailed understanding of altered prohormone processing in diabetes, potentially provide prognostic information, and possibly help quantify therapeutic responses.

The concept of proteoform-specific MS assays is based on the identification of a surrogate peptide unique to each target proteoform, the use of different proteolytic enzymes to generate such surrogate peptides, and the measurement of the peptides in specimens using LC-MS/MS after sample preparation. For instance, a surrogate peptide unique to the des-31,32 proinsulin could contain the C-peptide sequence plus the KR junction (Fig. 1C). One challenge for assay development is the extremely low abundance of prohormone proteoforms, which is often present in the pg/mL range (75). To address this sensitivity challenge, enrichment strategies (including the use of antibodies) are required prior to LC-MS/MS analyses. A key advantage of MS-based assays coupled with antibody-based enrichment is their ability to achieve both high sensitivity and specificity. The potential cross-reactivity of antibody reagents can be effectively overcome by the detection of surrogate peptides unique to each proteoform by MS. In this case, one or multiple antibodies can be used together to pull down all proteoforms of interest followed by LC-MS/MS-based multiplexed detection of surrogate peptides for each proteoform. In this strategy, it is critical to confirm consistent capture of all proteoforms since variations in binding characteristics across proteoforms could lead to biased quantitative results.

In an effort to develop a highly specific and reliable assay for proinsulin proteoforms in T1D research, TaMADOR has developed a prototype LC-MS/MS assay targeting surrogate peptides unique to each of the 4 proinsulin proteoforms (C-peptide, intact proinsulin, des-31,32, and des-64,65 proinsulin). An immunoaffinity enrichment step using an antibody cocktail is followed by Lys-C digestion and LC-MS/MS quantification of the surrogate peptides (Shen et al, manuscript in preparation). The method has a lower limit of quantification (LLOQ) in the 1- to 5-pg/mL range, marking the first reliable method for the quantification of multiple circulating proinsulin proteoforms. The utility of the assay was tested on serum samples from a cohort of adolescents with T1D and age-, sex-, and race-matched healthy controls (n = 20 for each group). The LC-MS/MS assay provided superior sensitivity and specificity compared to commercially available proinsulin immunoassays. While this LC-MS/MS assay for proinsulin proteoforms needs additional optimization, validation, and cross-lab assessment for transferability, it is anticipated that this assay will have broad utility in diabetes clinical studies, especially T1D, focusing on disease progression or therapeutic interventions.

In addition to the use of proteoforms to gauge β-cell function, there is also interest in the measurement of α-cell health, which is also compromised in diabetes (19, 20). One of the most important hormones produced by α-cells is glucagon, which is vital for proper insulin signaling and glucose homeostasis. In an effort to provide a specific assay for glucagon (and oxyntomodulin, which is produced from proglucagon by the intestine), TaMADOR developed an assay that uses antibodies to enrich these analytes after protein precipitation (Becker et al, manuscript under review). After validation of the assay, it was used to demonstrate that fasting patients with T1D have lower concentrations of plasma glucagon compared to healthy controls (Fig. 2D), but only when treated with an insulin pump. Patients using injectable insulin had plasma glucagon concentrations similar to healthy controls, suggesting that better glycemic control is associated with lower glucagon concentrations.

Finally, we should note one potential caveat of “proteoform-specific” LC-MS/MS assays that rely on the measurement of surrogate peptides, which results from the complexity of overlapping proteoforms. More specifically, while it is generally expected that there are 5 common proinsulin proteoforms (insulin, C-peptide, and intact, des-31,32, and des-64,65 proinsulin), a recent top-down proteomics study of mouse islets identified >100 insulin proteoforms potentially present at a wide range of relative concentrations (76). As a result, a specific surrogate peptide targeting a specific proteoform could inadvertently target unknown proteoforms that share the same surrogate peptide. Therefore, a surrogate peptide–centric LC-MS/MS assay should always be said to quantify the total amount of all possible proteoforms containing the same target peptide (77).

## Future Perspectives

Advances in biomarker discovery and the development of robust and accurate clinical assays will continue to play a significant role in diabetes clinical research, paving the way for better diagnostic tools and therapeutic approaches. Monitoring disease progression in patients through circulating molecular biomarkers will be crucial for understanding pathophysiology. However, the lack of circulating biomarkers that can accurately reflect endocrine (eg, β-cell and α-cell function) and exocrine mechanism and function, alongside a shortage of reliable and standardized assays, represent a major gap.

Biomarker discovery efforts using advanced proteomics and multi-omics approaches are likely to identify more novel biomarkers that will provide mechanistic information of therapeutic intervention and insights into the pathophysiology of diabetes (22, 25). The recent interest in islet prohormone processing in diabetes research (15, 26, 28) along with the complexity of prohormone proteoforms and the lack of proteoform-specific assays all converge on the significance that mass spectrometry could have in the development of proteoform-specific assays. MS-based discovery proteomics, including top-down proteomics, may also hold identify more novel proteoforms of interest to diabetes (76). Efforts to discover novel proteoforms as biomarkers, along with the development of robust proteoform-specific assays should enable a more detailed understanding of the importance of islet prohormone processing in pathophysiology and the early stages of diabetes progression.

The complexity of human samples and the indirect detection of protein analytes by the affinity reagents in immunoassays have made the standardization of current assays a long-standing challenge in the field, due to interfering substances common in human samples and nonspecific cross-reactivity of reagent antibodies with related molecules in serum and plasma samples (51, 52, 78). It is anticipated that MS-based assays will play an increasing role in assay standardization efforts by serving either as arbiters or reference methods. Furthermore, MS-based assays offer unique advantages for targeting specific proteoforms where developing affinity reagents specific to individual proteoforms is difficult due to a high degree of sequence similarity.

Improving the rigor and the reproducibility of clinical research is an important part of the NIDDK strategic plan. An example of this endeavor is represented by the HbA1c and C-peptide standardization programs (51, 73, 78). The TaMADOR projects will further enhance and facilitate future harmonization and standardization efforts. This aligns closely with the NIDDK's strategic goals by demonstrating the importance of collaboration in the development and deployment of reliable MS-based assays for novel biomarkers that enhance reproducibility in diabetes clinical research. The 75th anniversary of NIDDK provides a great opportunity to highlight the significant efforts within TaMADOR to improve rigor and reproducibility.

Although MS-based assays offer clear advantages, their widespread implementation in clinical labs remains challenging due to the complexity and cost of MS instrumentation. Nevertheless, it is noteworthy that clinical labs are increasingly adopting MS technology for diagnostic testing. For example, LC-MS assays are now routinely used in newborn screening (79, 80). Ongoing advances in LC-MS/MS instrumentation, with respect to sensitivity, throughput, and robustness, are making LC-MS/MS-based technologies more cost-effective and accessible for routine use. These improvements are set to enhance both translational research and clinical diagnosis/prognosis. Efforts in MS assay development, such as those within TaMADOR, will facilitate the translation of discoveries in biomarker research—such as the identification of novel proteoforms—into reliable and transferable MS-based proteoform-specific assays, thereby further empowering pathophysiological monitoring in clinical research. We foresee that continued investment and integration between MS assay development, implementation, and clinical research throughout intervention and longitudinal clinical studies will be essential to fully realize the potential of novel biomarker discovery and newly developed assays, ultimately leading to improved clinical outcomes.

## Data Availability

Data sharing is not applicable to this article as no datasets were generated or analyzed.

## Abbreviations

AAB: autoantibody
HbA1c: glycated hemoglobin
IAPP: islet amyloid polypeptide
LC-MS/MS: liquid chromatography–tandem mass spectrometry
MS: mass spectrometry
NIDDK: National Institute of Diabetes and Digestive and Kidney Diseases
T1D: type 1 diabetes
T2D: type 2 diabetes
